# Case report: Utility, complications, and short-term outcomes in three dogs managed with percutaneous pigtail cystostomy catheters for urethral obstruction

**DOI:** 10.3389/fvets.2023.1200406

**Published:** 2023-08-10

**Authors:** Yanshan Er, Meghan E. Fick, Erin Long Mays

**Affiliations:** Department of Veterinary Clinical Medicine, University of Illinois at Urbana-Champaign, Urbana, IL, United States

**Keywords:** pigtail, cystostomy, catheter, urinary, diversion, obstruction

## Abstract

**Objective:**

This study aimed to describe the utility, complications, and short-term outcomes of three dogs managed with percutaneous pigtail cystostomy catheters placed in the emergency room (ER).

**Case summary:**

Three dogs were presented separately to the ER for unalleviated mechanical urethral obstruction secondary to urolithiasis and urethral neoplasia. Retrograde urinary catheterization and urohydropulsion were not successful after multiple attempts. Percutaneous pigtail cystostomy catheters were placed under sedation to achieve temporary urinary diversion, and were successful in two of the three dogs. Complications encountered include mild abdominal effusion, unsuccessful placement resulting in hemorrhagic abdominal effusion, steatitis, abdominal pain, and kinking of the catheter. The two dogs diagnosed with urolithiasis were discharged from the hospital, and the dog diagnosed with urethral neoplasia was humanely euthanized due to poor prognosis.

**New or unique information provided:**

When successful, the placement of pigtail cystostomy catheters allowed for temporary urinary diversion until definitive treatment could be performed and were well tolerated. Short-term outcomes were good. Complications arising from this procedure were common and increased morbidity. The risk of unsuccessful catheter placement may be increased when the procedure is performed in an over conditioned patient or by an inexperienced operator. Careful case selection and risk–benefit analysis should be considered before attempting this procedure. Further studies are necessary to evaluate the ideal technique, incidence of complications, and outcomes of this procedure.

## 1. Introduction

Canine mechanical urethral obstruction is commonly encountered in the emergency room (ER). The main causes include urolithiasis, neoplasia, trauma, and stricture of the lower urinary tract (1). First-line management typically involves achieving temporary urinary diversion via retrograde urinary catheterization until definitive treatment such as surgery, lithotripsy, or cystoscopy can be performed. When unsuccessful, alternative techniques for temporary urinary diversion include intermittent decompressive cystocentesis or surgical cystostomy tube placement (2, 3). These options can be associated with greater nursing burden and patient discomfort, or anesthesia and surgical expertise, respectively. A technique for ultrasound-guided percutaneous placement of a pigtail cystostomy catheter has been described to achieve temporary urinary diversion in a dog (4). The utility and complications associated with this procedure have been described in cats (5) but not in dogs. This report describes the utility, complications, and short-term outcomes of three dogs managed with percutaneous pigtail cystostomy tubes placed in the ER for unalleviated mechanical urethral obstruction.

## 2. Case presentation

### 2.1. Case 1

A 13-year-old female spayed Dachshund weighing 7.8 kg (BCS 5/9) was presented to a tertiary referral hospital for mechanical urethral obstruction. On the day of presentation, she was evaluated by her referring veterinarian for a 48-h history of unproductive stranguria and a large, turgid bladder when palpated. Abdominal radiographs did not reveal any radiopaque uroliths. On presentation to the referral hospital, the dog was estimated to be 5% dehydrated and had a firm, turgid, inexpressible bladder and mucohemorrhagic vulvar discharge. Rectal examination revealed urethral thickening with suspicions of a urethral mass. Packed cell volume (PCV) was 48%, and total solids (TS) was 8.4 g/dL. Complete blood count (CBC) showed mild leukocytosis (22.7 K/uL, reference interval [RI] 6.00–17.00 K/uL) with a mature neutrophilia (20.43 K/uL, RI 3.00–11.50 K/uL). Serum chemistry was clinically unremarkable. Standardized four-quadrant (diaphragmaticohepatic, hepatorenal, cystocolic, and splenorenal) abdominal-focused assessment with sonography for trauma (aFAST) revealed no peritoneal effusion.

**Figure 1:**
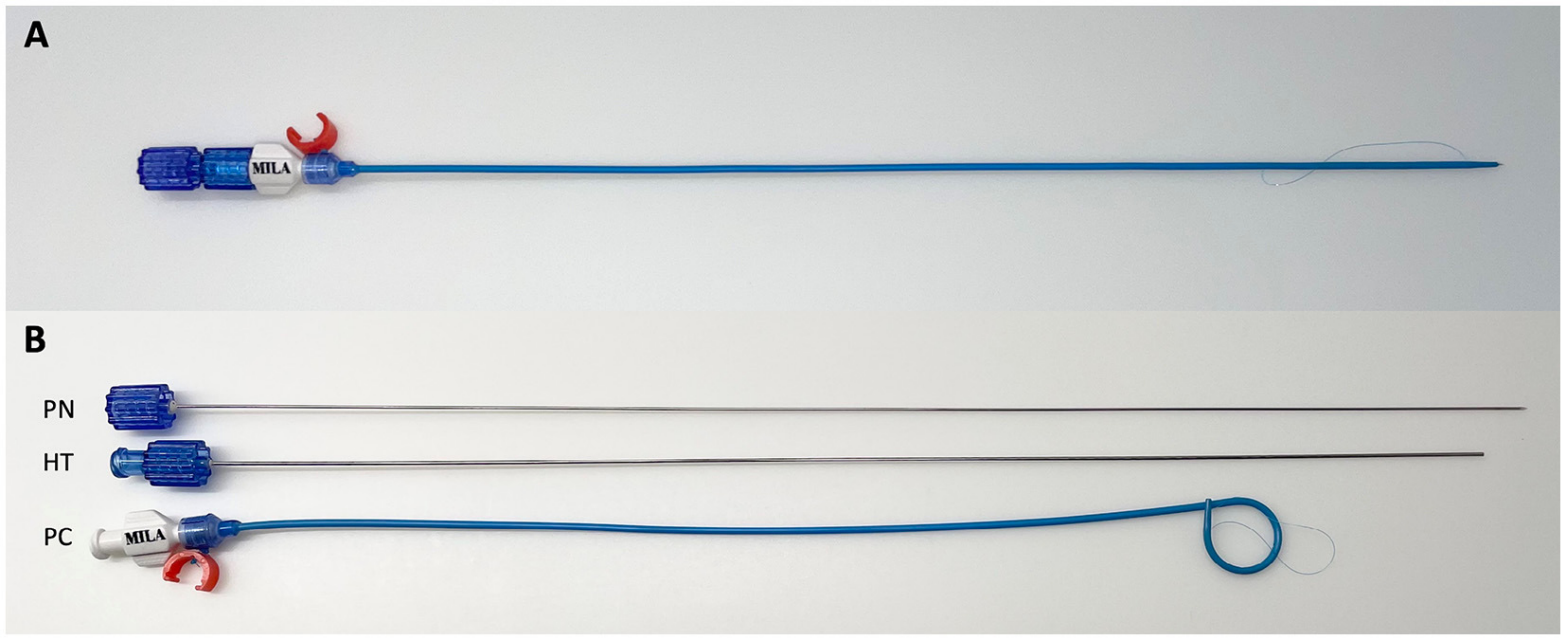
Assembled pigtail catheter **(A)**. Pigtail catheter disassembled **(B)** to show its components comprising of the puncture needle (PN), hollow trochar (HT), and pigtail catheter (PC).

**Figure 2:**
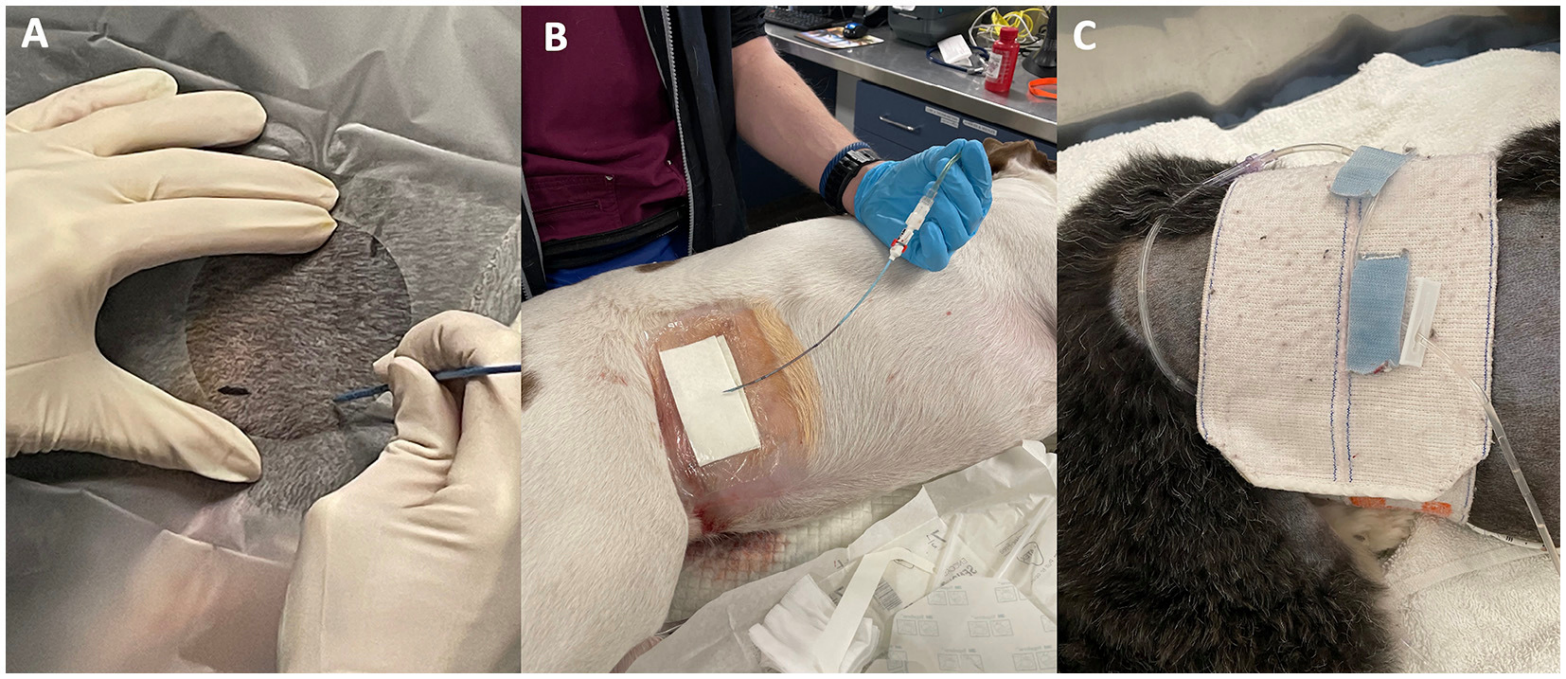
Pigtail catheter placement using the right lateral approach. An operator demonstrating intended puncture site where imaginary lines of the permanent marker meet over the abdomen **(A)**, cystostomy site covered by sterile dressing **(B)**, and catheter tubing secured to the abdomen with a Velcro abdominal binder **(C)**.

The patient was sedated with methadone (0.26 mg/kg IV), midazolam (1 mg/kg IV), and dexmedetomidine (3.8 mcg/kg IV) and then placed in sternal recumbency. The retrograde placement of a 6 Fr Foley urinary catheter (Foley catheter with wire stylet, MILA International, Florence, South Carolina, US) was attempted several times unsuccessfully due to an obstruction 3–4 mm into the urethra. After consultation with specialty services, it was elected to achieve temporary urinary diversion with a pigtail cystostomy catheter (Pigtail catheter, MILA International, Florence, South Carolina, US) (Figure 1) until additional diagnostics could be performed the next day. The patient was sedated with additional dexmedetomidine (2 mcg/kg IV) and repositioned in left lateral recumbency. A wide clip of the right caudolateral abdomen was performed. The distended bladder was palpated, and the intended puncture site close to the bladder neck was identified with ultrasound guidance, which was then marked with a permanent marker outside of the direct puncture site. The surgical field was aseptically prepped with chlorhexidine scrub and alcohol and then covered with a sterile, semi-opaque fenestrated drape. A stab incision was made through the skin with a #11 blade. The non-dominant hand was used to immobilize the bladder, and a 6 Fr pigtail cystostomy catheter, held in the dominant hand, was advanced into the abdomen until urine was observed to flow from the catheter. The puncture needle was then removed. The hollow trochar and cystostomy catheter were advanced a few millimeters before the cystostomy catheter was completely advanced into the bladder, and the hollow trochar was removed. The pigtail loop was tightened by pulling the locking string taut and securing it around the proximal end of the cystostomy catheter. Ultrasound visualization of the locked loop seated within the bladder lumen was confirmed. The catheter was then connected to a closed urine collection set. The stoma site was covered with a waterproof film dressing with a non-adherent pad (Tegaderm + Pad, 3M). The urine collection set tubing was then secured to the patient's abdomen with a velcro abdominal binder (Prody^TM^ abdominal binder with drain fasteners, Bird & Cronin) (Figure 2).

The patient was admitted to the intensive care unit and kept on balanced isotonic fluid (Normosol^®^-R, Hospira Inc.) (60 ml/kg/day plus 5 % rehydration corrected over 24 h IV), methadone (0.1 mg/kg IV q6h), and ampicillin–sulbactam (30 mg/kg IV q8h). Caring measures for the cystostomy catheter involved swabbing the urine bag drainage port and the cystostomy catheter (proximally to distally) with 0.05% chlorhexidine solution prior to quantifying urine q6h. The cystostomy insertion site was inspected and cleaned with 0.05% chlorhexidine solution q24h, and the dressing was changed at the same time or more frequently if strikethrough was noted.

**Figure 3:**
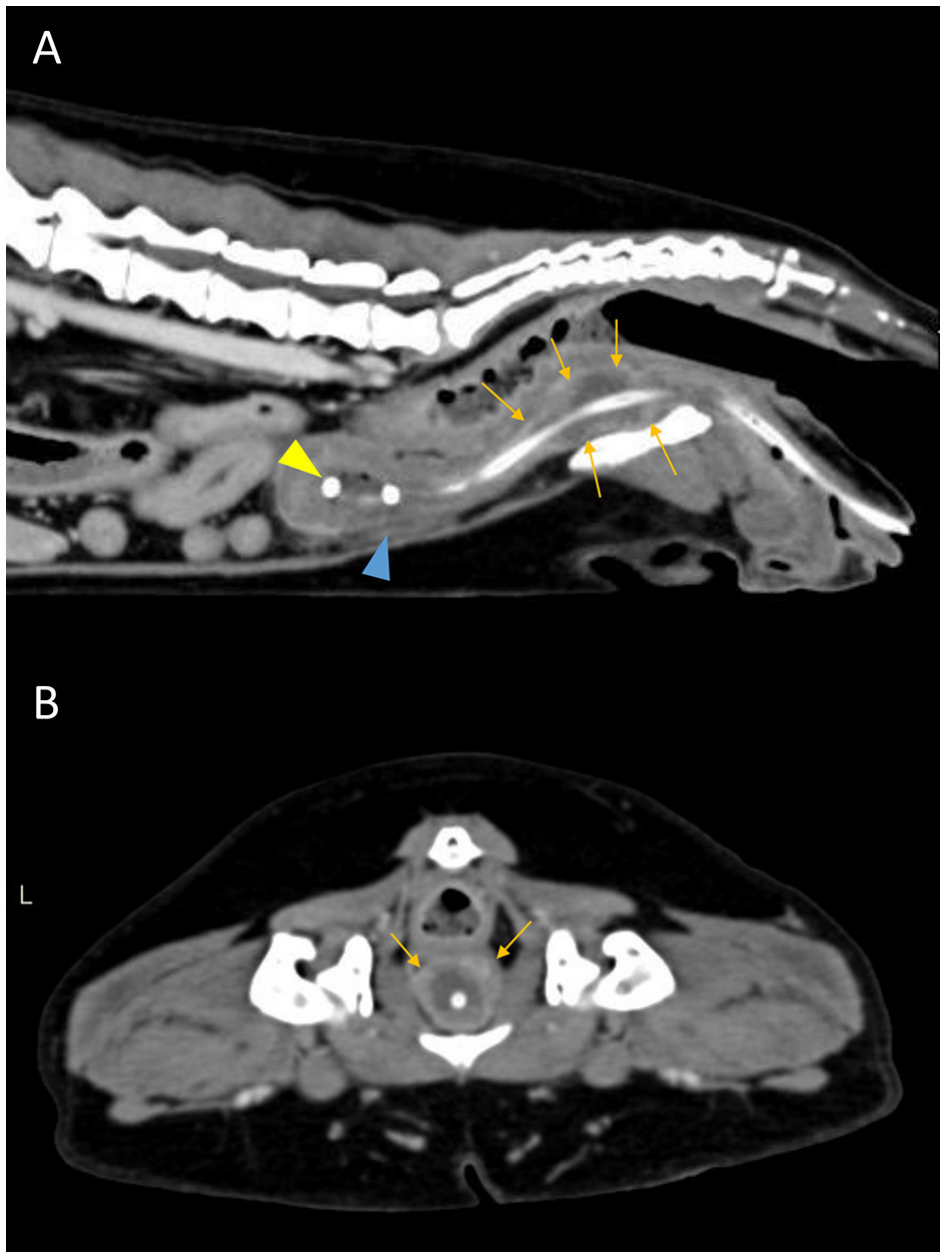
Postcontrast sagittal **(A)** and transverse **(B)** CT images showing a diffuse urethral mass (orange arrows). The pigtail loop of the cystostomy catheter can be seen within the bladder lumen (yellow arrowhead). There is mild abdominal effusion present (blue arrowhead).

The next day, the patient was sedated for a computed tomography (CT) scan and diagnosed with diffuse urethral neoplasia. Mild abdominal effusion was observed around the pigtail catheter (Figure 3). Conservative medical management was elected, and piroxicam (0.26 mg/kg PO q24h) and prazosin (0.13 mg/kg q8h) were initiated. The pigtail cystostomy catheter remained in the patient during the 63-h duration of hospitalization, with the daily urine output in the normal range of 1.1 to 1.46 ml/kg/h. She maintained a good appetite and was assessed to be comfortable with a consistent score of 0 out of 24 on the short-form Glasgow Composite Measure Pain Scale (CMPS-SF) performed q6h (6). She walked q6h with the cystostomy catheter in place without dislodgement or kinking of the catheter. Because the dog was clinically stable and palliatively managed, an investigation into the mild abdominal effusion was not pursued.

The cystostomy catheter was clamped 44-h post-admission to allow for bladder filling and to assess the patient's ability to urinate. Serial standard aFAST was performed 6- and 12-h post-occlusion of the catheter to monitor for progressive bladder distension. No abdominal effusion was reported by two different operators. Over the next 16 h, the patient was walked q4h and exhibited severe stranguria, only dribbling small amounts of urine. She was humanely euthanized with pentobarbital sodium (250 mg/kg IV) due to poor response to treatment.

### 2.2. Case 2

An 8-year-old intact male mixed breed dog weighing 29.2 kg (BCS 7/9) was presented to a tertiary referral hospital for mechanical urethral obstruction. He was initially presented to his primary care veterinarian on the same day for a 1-week history of stranguria and hematuria. Abdominal radiographs revealed multiple cystoliths, urethroliths, and severe prostatomegaly. Retrograde urohydropulsion was unsuccessful, prompting referral. A decompressive cystocentesis was performed prior to transfer.

On presentation, the patient was quiet, alert, and responsive. Physical examination revealed a tense, painful abdomen with a distended, inexpressible bladder and severe prostatomegaly. Rectal examination revealed symmetrical prostatomegaly, and no stones were palpated in the urethra. Venous blood gas analysis showed mild acidemia (pH 7.333, RI 7.39–7.49), mild hyperlactatemia (3.2 mmol/L, RI 0.435–2.93 mmol/L), and mild azotemia (creatinine 1.7 mg/dL, RI 0.73–1.19 mg/dL; BUN 45 mg/dL, RI 9.1 – 24.5 mg/dL). PCV was 52%, and total solids was 8.4 g/dL. CBC showed mild leukocytosis (22.44 K/uL), mature neutrophilia (18.4 K/uL), and moderate monocytosis (2.47 K/uL, RI 0.20–1.40 K/uL). Serum chemistry showed mild azotemia (creatinine 1.8 mg/dL, BUN 64 mg/dL), mild hyperproteinemia (7.3 g/dL, RI 5.1–7.0 g/dL), mild hyperphosphatemia (5.4 mg/dL, RI 2.7–5.2 mg/dL), and severe CK elevation (1942 U/L, RI 26–310 U/L). The urinalysis of a cystocentesis sample showed dilute urine (USG 1.017), protein 3+, glucose 1+, blood 3+, and RBC > 100/hpf. Electrocardiography revealed a second-degree atrioventricular block, infrequent ventricular escape beats, and a heart rate of 116 beats per minute. aFAST was negative for abdominal effusion.

The patient was sedated with methadone (0.2 mg/kg IV), midazolam (0.2 mg/kg IV), and alfaxalone (0.25 mg/kg IV) for retrograde urohydropulsion. A 6 Fr Foley catheter and an 8 Fr Red Rubber catheter (Red Rubber urethral catheter, Medline Industries Inc., IL, US) were used, but multiple attempts were unsuccessful. It was elected to achieve temporary urinary diversion with a pigtail cystostomy catheter until surgery could be performed.

The placement of an 8 Fr pigtail cystostomy catheter was attempted by a first operator using the same technique as described in Case 1 but using a left lateral approach since the dog was already in right lateral recumbency. Resistance was met as the catheter was introduced through the skin incision, resulting in the bending of the puncture needle and trochar. Another attempt was made through the same skin incision but was unsuccessful. A second, more experienced operator took over but encountered the same complication. Further attempts were aborted due to concern for trauma to the abdominal viscera. A new pigtail catheter was used during each attempt. Recheck aFAST showed a moderate volume of abdominal fluid in the cystocolic quadrant, and a diagnostic abdominocentesis yielded hemorrhagic effusion with a PCV of 23% and total solids of 4.0 g/dL. An ultrasound-guided decompressive cystocentesis was performed, and approximately 300 ml of hemorrhagic urine was obtained.

Recovery was unremarkable, and the patient was transferred to the intensive care unit. Balanced isotonic solution (LRS, Hospira Inc.) was administered at 40 ml/kg/day IV in addition to methadone (0.2 mg/kg IV q6h), gabapentin (10.9 mg/kg PO q8h), and trazodone (3.4 mg/kg PO q8-12h PRN). Repeat aFAST at 3-h intervals performed by the same second operator showed subjectively static abdominal effusion in the cystocolic quadrant. The patient's vital parameters were monitored q6h and remained within normal limits; however, he received rescue methadone (0.2 mg/kg IV) early, 4 h after the prior dose due to abdominal pain (CMPS-SF 7 out of 24). A decompressive cystocentesis was performed 8 h after the first cystocentesis was performed on the palpation of a turgid bladder.

Eight hours post admission, PCV and TS were decreased at 43% and 7.2 g/dL, respectively. Venous blood gas analysis showed improved azotemia (creatinine 1.6 mg/dL, BUN 38 mg/dL). The patient was anesthetized (premedication: methadone 0.2 mg/kg IV, induction: propofol 2 mg/kg IV and lidocaine 2 mg/kg IV, maintenance: inhaled isoflurane 0.5–2%). Atracurium (0.07 mg/kg) and lidocaine (0.7 mg/kg) were instilled into the urethra using an 8 Fr Red Rubber catheter. Retrograde urohydropulsion was successful. Intraoperatively, edematous, and hyperemic fat was observed on the ventral aspect of the bladder, along with 200–300 ml of hemorrhagic abdominal fluid. The bladder appeared markedly thickened and hyperemic. No trauma to the bladder wall from pigtail cystostomy attempts was visualized. A routine cystostomy and scrotal orchiectomy were performed. The stab incision in the skin made during the pigtail cystostomy attempts was repaired with skin sutures.

The dog was tachycardic immediately postoperatively (HR 180 bpm). This did not improve with a fluid bolus (LRS 10ml/kg IV), and the patient was painful on abdominal palpation with a CMPS-SF of 7 out of 24. Analgesia was escalated from intermittent methadone (0.2 mg/kg IV q6h) to a fentanyl CRI (3 mcg/kg/hr) and ketamine CRI (3 mcg/kg/min). These infusions were discontinued 15 h postoperatively on the resolution of tachycardia and abdominal pain. The dog was discharged 48 h postoperatively. Follow-up phone calls 1- and 19-days postoperatively confirmed that the dog was doing well with no concerns urinating.

### 2.3. Case 3

A 3-year-old male castrated pit bull mix weighing 35 kg (BCS 5/9) was presented to a tertiary referral hospital for mechanical urethral obstruction. He was initially presented to his referring veterinarian for a 24-h history of vomiting and worsening stranguria. Abdominal radiographs confirmed cystoliths and urethroliths. The patient was sedated, and retrograde urohydropulsion was attempted but unsuccessful. A decompressive cystocentesis was performed prior to transfer.

On presentation to the referral hospital, the patient's vital parameters were within normal limits. A full, soft but inexpressible bladder was palpated. CBC was unremarkable. Serum chemistry was notable for moderate azotemia (creatinine 2.2 mg/dL, BUN 34 mg/dL).

The patient was sedated with methadone (0.2 mg/kg IV), dexmedetomidine (4 mcg/kg IV), and alfaxalone (2 mg/kg IV to effect). Retrograde urohydropulsion was attempted using Red Rubber and Foley catheters of unspecified sizes and was unsuccessful. The placement of a pigtail cystostomy catheter was elected to achieve temporary urinary diversion until surgery could be performed at a later time. The patient was already in left lateral recumbency, so a right lateral approach was performed as described in Case 1 and was completed without any complications.

The patient was admitted to the intermediate care ward on recovery and placed on LRS (40 ml/kg/day IV) and methadone (0.2 mg/kg IV q6h), along with cystostomy catheter care, as described in Case 1. He was walked q6h and had a consistent CMPS-SF score of 0 out of 24 every q6h while the cystostomy catheter was in place. The collection set line was found to be kinked once during hospitalization after no urine was observed in the collection bag during a scheduled treatment hour. Urine flow was reestablished after unkinking the line. Repeat serum chemistry performed 15 h after admission showed resolution of azotemia (creatinine 1.1 mg/dL, BUN 14 mg/dL).

**Figure 4:**
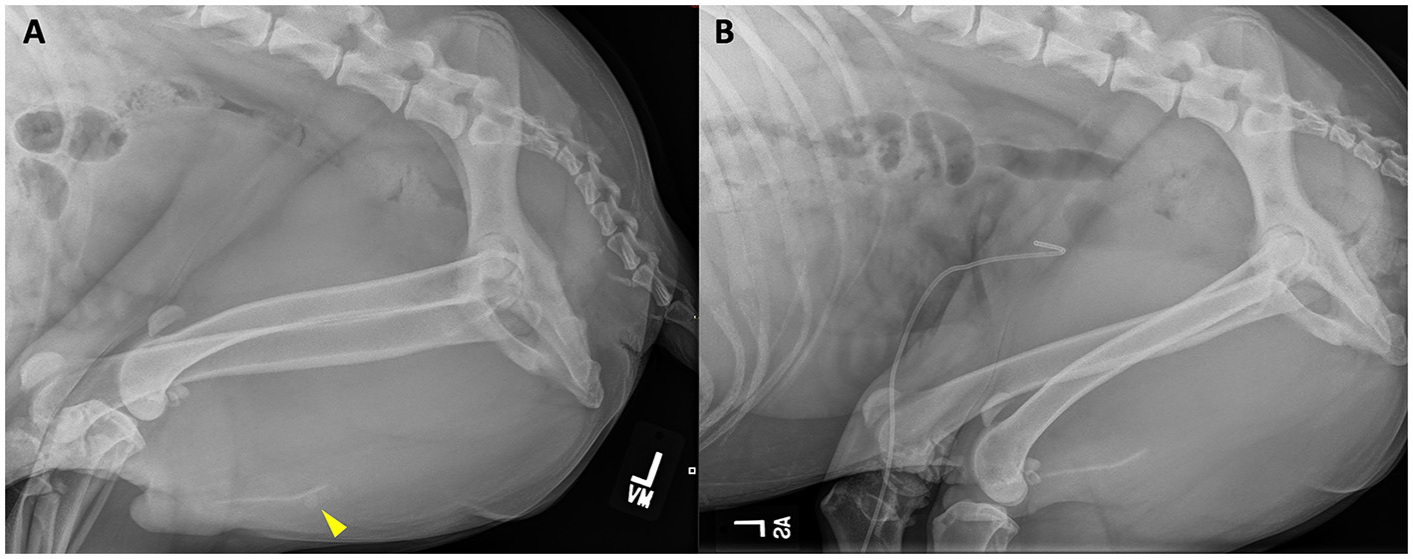
Left lateral abdominal radiograph prior to **(A)** retrograde urohydropulsion of urethral stones (yellow arrowhead) and after successful retrograde urohydropulsion **(B)**. The previous urethral stones are no longer present at the level of the os penis. A radiopaque pigtail catheter is seated within the bladder lumen.

The patient was anesthetized for surgery 44 h post-admission (premedication: methadone 0.29 mg/kg IV, induction: propofol 3.4 mg/kg IV and lidocaine 2 mg/kg IV, maintenance: inhaled isoflurane 1.25–1.5%). A lumbosacral epidural was administered with bupivacaine (0.5 mg/kg). Successful retrograde urohydropulsion was performed with an 8 Fr Red Rubber catheter (Figure 4). A routine cystotomy followed. No abdominal effusion or trauma to the abdominal viscera were visualized intraoperatively. The cystostomy insertion site in the bladder had sealed without requiring repair. The patient was discharged 24 h postoperatively. A follow-up phone call 1-week following discharge confirmed that the dog was urinating normally.

## 3. Discussion

In this case series, pigtail cystostomy catheters were used as a second-line intervention only after several failed attempts at retrograde urinary catheterization. In the dogs with obstruction secondary to urethrolithiasis, retrograde urohydropulsion was performed with per-rectum occlusion of the urethra proximal to the suspected obstruction site (7) to increase the chance of success.

Achievement of temporary urinary diversion in Cases 1 and 3 allowed for the prevention of severe electrolyte and acid–base disturbances, a more rapid correction of dehydration with IV fluid therapy (8), increased patient comfort, eliminated the repeated risk and burden on nursing staff associated with intermittent decompressive cystocenteses, and allowed further diagnostics and therapeutics to be performed at a later stage. This is particularly helpful when the required manpower or treatment options such as cystoscopy and lithotripsy are unavailable. Relief of backpressure from the bladder may have increased the success of retrograde urinary catheterization. Successfully placed cystostomy catheters did not interfere with dogs' daily activities. The use of a pigtail catheter allowed for the assessment of urethral patency in the case of obstructive urethral neoplasia. Pigtail cystostomy catheters were placed in adequately sedated patients, and anesthesia was not necessary. Bladder rupture, a complication of trauma to a distended bladder (9), was not observed despite the relatively large diameter of cystostomy tubes used.

Cats that had similar pigtail cystostomy catheters placed were found to have a 40% complication rate, including dislodgement, urine leakage, urinary tract infection, pyrexia, and bladder rupture (5). Most complications were minor and did not affect quality of life. Surgically placed tube cystostomies in dogs and cats were found to have a 49% complication rate although these tubes were in place for a longer period (median indwelling time of 11 days) (10). Complications encountered in our case series were common and included mild abdominal effusion, failed cystostomy catheter placement resulting in steatitis and abdominal pain, mild intraabdominal hemorrhage, and kinking of the catheter. In Case 1, further investigation into the abdominal effusion detected on CT was not pursued, but the possibility of catheter leakage cannot be excluded and should warrant further diagnostic testing. aFAST and CT scans have moderate-to-excellent agreement in the detection of peritoneal effusion although this is operator dependent and aFAST was performed by different operators in Case 1 (11). Mild perivesicular abdominal effusion is common in patients with prolonged urethral obstruction (12, 13) and was not differentiated from leakage from the cystostomy catheter. The potential for seeding of neoplastic cells or the development of urosepsis is an additional consideration. In Case 2, iatrogenic abdominal trauma increased the analgesic requirement and prolonged the hospital stay. Failed attempts took up considerable time, which is counterproductive in a busy ER. In Case 3, kinking of the tubing prevented accurate quantification of urine, which is important in guiding fluid therapy.

An ideal technique for the placement of percutaneous pigtail cystostomy catheters has not been established. Dogs were placed in right or left lateral recumbency depending on clinician preference. Ultrasound guidance was used to identify the bladder prior to puncture but not during, which may have increased the risk of trauma to other organs. The bladder was punctured close to its neck to minimize pressure on the bladder wall with apical bladder filling but could increase the risk of ureteral and prostatic trauma. Future studies comparing outcomes associated with various placement locations could help define the optimal approach with the fewest complications. The dog in Case 2 was overweight, and excess subcutaneous tissue could have prevented the catheter from puncturing the bladder. The first operator in Case 2 did not have prior experience in the procedure, which may have contributed to the unsuccessful attempt. Tubes were not sutured to the abdomen as described in previous studies (4, 5), which could have resulted in kinking, increased movement, and subsequent urine leakage.

Limitations of this case series include a lack of investigation into the abdominal effusion in Case 1, missing information in the medical record of incisional site complications (subcutaneous edema, stoma leakage, and infection) and the time taken to place the pigtail cystostomy catheters, variability in placement technique, and operator experience. Long-term outcomes were not evaluated as dogs had indwelling cystostomy catheters for a range of only 44–60 h. Risks such as catheter-associated infection (8) were not evaluated.

While the placement of percutaneous pigtail cystostomy catheters is a feasible option for temporary canine urinary diversion in unalleviated mechanical urethral obstructions presenting to the ER, complications encountered were frequent and should be discussed with owners prior to attempts. This technique should be reserved for patients having or at risk of developing life-threatening electrolyte or acid–base disturbances and when definitive treatment is not available in a timely manner. Care should be taken when performing this procedure in patients with increased body habitus. Experience in the procedure and ultrasound guidance will likely increase the successful placement of catheters. As this procedure becomes more widely performed, larger-scale studies should be done to evaluate the ideal technique, incidence of complications, and long-term outcomes.

## Data availability statement

The original contributions presented in the study are included in the article/supplementary material, further inquiries can be directed to the corresponding author.

## Ethics statement

Ethical approval was not required for this animal study because this is a case report and the medical records were reviewed retrospectively. No experimental procedures were performed. A hospital consent form was signed by the owners of the animals in this case report, consenting to all diagnostics and treatment interventions performed. Written informed consent was obtained from the owners for the publication of this case report.

## Author contributions

YE clinical management of the cases, initial draft, critical revisions, and final manuscript approval. MEF and ELM critical revisions and final manuscript approval. All authors contributed to the article and approved the submitted version.

## References

[1] Rieser TM. Urinary tract emergencies. Vet Clin N Am. (2005) 35:359–73. 10.1016/j.cvsm.2004.12.001 15698915

[2] Stiffler KS McCrackin Stevenson MA Cornell KK Glerum LE Smith JD Miller NA. Clinical use of low-profile cystostomy tubes in four dogs and a cat. J Am Vet Med Assoc. (2003) 223:325–9. 10.2460/javma.2003.223.325 12906227

[3] Salinardi BJ Marks SL Davidson JR Senior DF. The use of a low-profile cystostomy tube to relieve urethral obstruction in a dog. J Am Anim Hosp Assoc. (2003) 39:403–5. 10.5326/0390403 12873032

[4] Culler CA Fick ME Vigani A. Ultrasound-guided placement of pigtail cystostomy tubes in dogs with urethral obstruction. J Vet Emerg Crit Care. (2019) 29:331–6. 10.1111/vec.12832 30994963

[5] Nurra G Howes C Chanoit G Meakin L Parsons K Friend E. Clinical use and complications of percutaneous cystostomy pigtail catheters in 25 cats. J Feline Med Surg. (2022) 24:e28–33. 10.1177/1098612X221080902 35363097 PMC9161432

[6] Reid J Nolan AN Pawson P Hughes JML Lascelles D Scott EM. Development of the short-form Glasgow composite measure pain scale (CMPS-SF) and derivation of an analgesic intervention score. Anim Welf. (2007) 16:97–104. 10.1017/S096272860003178X28514527

[7] Aldritch J. Urethral catheterization. In:Burkitt Creedon JM Davis H, editors. Advanced Monitoring and Procedures for Small Animal Emergency and Critical Care. Chichester, WS: John Wiley & Sons, Ltd. (2012), 395–408.

[8] Ostroski C Clarke DL. Urinary Diversion in the Emergency Room. In:Drobatz KJ Hopper K Rozanski EA Silverstein DC, editors. Textbook of Small Animal Emergency Medicine. Hoboken, NJ: John Wiley & Sons, Ltd. (2018) 673–679. 10.1002/9781119028994.ch106

[9] Stafford JR Bartges JW. A clinical review of pathophysiology, diagnosis, and treatment of uroabdomen in the dog and cat. J Vet Emerg Crit Care. (2013) 23:216–29. 10.1111/vec.12033 23470168

[10] Beck AL Grierson JM Ogden DM Hamilton MH Lipscomb VJ. Outcome of and complications associated with tube cystostomy in dogs and cats: 76 cases (1995-2006). J Am Vet Med Assoc. (2007) 230:1184–9. 10.2460/javma.230.8.1184 17501659

[11] Walters AM O'Brien MA Selmic LE Hartman S McMichael M O'Brien RT. Evaluation of the agreement between focused assessment with sonography for trauma (AFAST/TFAST) and computed tomography in dogs and cats with recent trauma. J Vet Emerg Crit Care. (2018) 28:429–35. 10.1111/vec.12732 29901282

[12] Nevins JR Mai W Thomas E. Associations between ultrasound and clinical findings in 87 cats with urethral obstruction. Vet Radiol Ultrasound. (2015) 56:439–47. 10.1111/vru.12259 25850697

[13] Gerken KK Cooper ES Butler AL Chew DJ. Association of abdominal effusion with a single decompressive cystocentesis prior to catheterization in male cats with urethral obstruction. J Vet Emerg Crit Care. (2020) 30:11–7. 10.1111/vec.12914 31840942

